# Evaluation of lymphocytic infiltration in the bronchial glands of Sjögren’s syndrome in transbronchial lung cryobiopsy

**DOI:** 10.1186/s12890-020-01318-0

**Published:** 2020-10-23

**Authors:** Hiroko Okabayashi, Tomohisa Baba, Ryota Ootoshi, Ryota Shintani, Erina Tabata, Satoshi Ikeda, Takashi Niwa, Tsuneyuki Oda, Ryo Okuda, Akimasa Sekine, Hideya Kitamura, Shigeru Komatsu, Eri Hagiwara, Tamiko Takemura, Takuro Sakagami, Takashi Ogura

**Affiliations:** 1 Department of Respiratory medicine, Kanagawa Cardiovascular and Respiratory Center, 6-16-1 Tomioka-Higashi, Kanazawa-ku, Yokohama city, Kanagawa 236-0051 Japan; 2 Department of Respiratory Medicine, Kumamoto University Hospital, Faculty of Life Sciences, Kumamoto University, 1-1-1 Honjo, Chuo-ku, Kumamoto, 860-8556 Japan; 3 Department of Pathology, Kanagawa Cardiovascular and Respiratory Center, 6-16-1 Tomioka-Higashi, Kanazawa-ku, Yokohama city, Kanagawa 236-0051 Japan

**Keywords:** Sjögren’s syndrome, Bronchial gland, Lymphocytic infiltration, Transbronchial lung cryobiopsy

## Abstract

**Background:**

Sjögren’s syndrome (SS) is a systemic autoimmune disease characterized by deteriorated exocrine gland function with associated lymphocytic infiltration. However, there are few pathological studies on bronchial glands in SS. In this study, we aimed to clarify pathological features of bronchial glands in SS.

**Methods:**

We retrospectively evaluated infiltration of lymphocytes in the bronchial glands incidentally collected by transbronchial lung cryobiopsy (TBLC), which were performed for the diagnosis of diffuse lung diseases. The degrees of lymphocyte infiltration in the bronchial glands were classified into four grades (grade 0–3). We compared the degrees of infiltration of SS with those of other diffuse lung diseases.

**Results:**

TBLC for diagnosis of diffuse lung diseases were performed on 432 cases during the study period. The samples of 50 cases included bronchial glands. Of those, 20 cases were excluded due to insufficient size or influence of therapy. The remaining 30 cases included 17 of idiopathic interstitial pneumonias, 5 of chronic hypersensitivity pneumonia, 6 of connective tissue disease (SS; *n* = 4, systemic sclerosis; *n* = 1, dermatomyositis; *n* = 1) and 2 of other diseases. In SS, infiltration of lymphocytes was observed in all cases; grade 1 in one, grade 2 in one, and grade 3 in two cases. In contrast, 11 of 26 in other diseases showed no lymphocytes infiltration, with the remaining 15 of grade 1 infiltration. Grade 2 or more infiltration were found only in SS but not in other diseases.

**Conclusion:**

Our results suggested that high-grade lymphocytic infiltration of bronchial glands is a distinct characteristics in SS.

## Background

Sjögren’s syndrome (SS) is a systemic autoimmune disease characterized by deteriorated salivary and lacrimal gland function with lymphocytic infiltration of exocrine glands. Not only salivary and lacrimal glands but also various extraglandular organ systems such as lung and kidney are affected in SS. Bronchial glands are morphologically similar to salivary glands. Salivary gland biopsy is a technique broadly applied for the diagnosis of SS [1–3]. High-grade lymphocyte infiltration in salivary gland is observed in SS. However, there are very few literatures that describe the characteristics of cell infiltration and histopathological changes in the bronchial glands of SS.

Recently, the utility of transbronchial lung cryobiopsy (TBLC) has been reported in the diagnosis of diffuse lung disease [4–7]. Cryoprobe-retrieved specimens are larger than those of transbronchial forceps biopsies and less crush. TBLC tend to sample more proximal portion of the lung apart from the pleural than surgical lung biopsy (SLB). Although bronchial glands are rarely collected by SLB or transbronchial forceps biopsy, they are sometimes incidentally biopsied by TBLC. In this study, we aimed to clarify whether high-grade lymphocytic infiltration in the bronchial glands was observed as a distinct feature in SS.

## Methods

### Patients

**Fig. 1:**
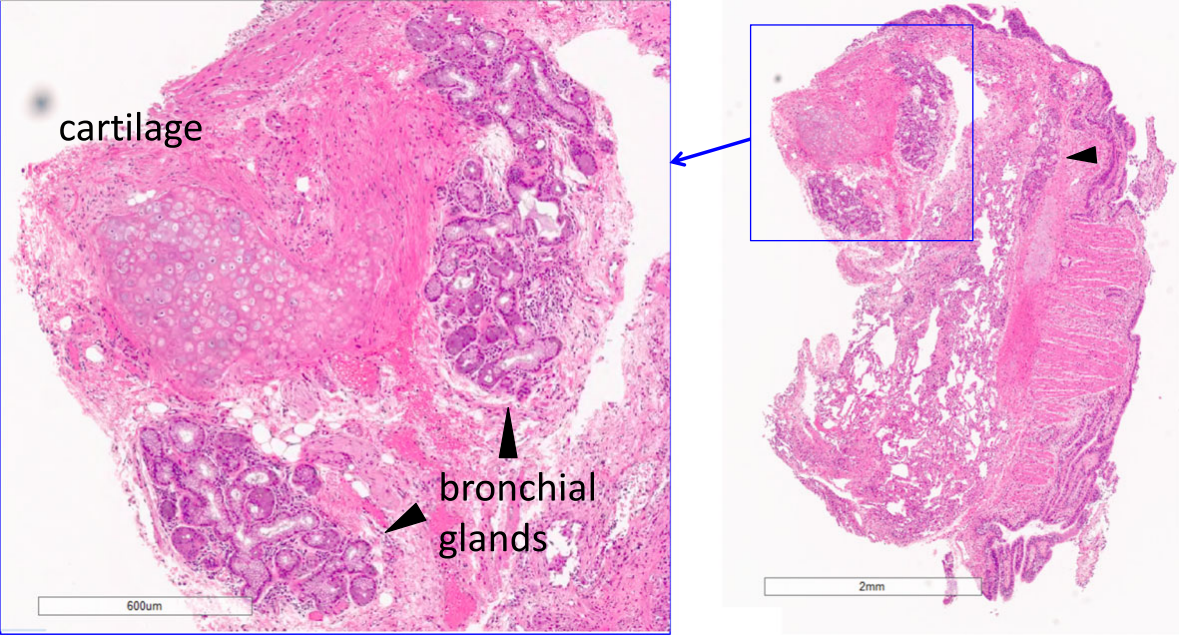
Bronchial glands that were collected by transbronchial lung cryobiopsy. The bronchus with cartilage was biopsied. Head arrows show bronchial glands

We retrospectively reviewed all the specimens collected by TBLC, which were performed for the diagnosis of diffuse lung diseases between May 2017 and October 2018 in Kanagawa Cardiovascular and Respiratory Center. Among those, specimens incidentally including the bronchial glands were extracted (Fig. 1). The exclusion criteria of this study were as follows: (1) the size of biopsied bronchial glands was small (< 0.05mm^2^); (2) medication such as steroids or immunosuppressant have already been given before biopsy. Institutional review board of Kanagawa Cardiovascular and Respiratory Center approved the study protocol (KCRC-19-0032).

### The procedure of transbronchial cryobiopsy

The patients were intubated with flexible endotracheal tube using moderate to deep sedation. Sedative agents were midazolam plus fentanyl. Balloon blocker (Edwards Lifesciense, Fogarty E-80-4F) was routinely placed balloon blocker in the targeted sub-segmental bronchi. A 1.9 mm or 2.4 mm cryoprobe (Erbe Elektromedizin GmBH) was introduced through the working channel of a flexible bronchoscope under fluoroscopic guidance into the selected bronchi. Freezing time of cryoprobe was 6–7 s with 1.9 mm probe and 4–5 s with 2.4 mm probe.

### Scoring of lymphocytes and plasma cells infiltration in bronchial glands

**Fig. 2:**
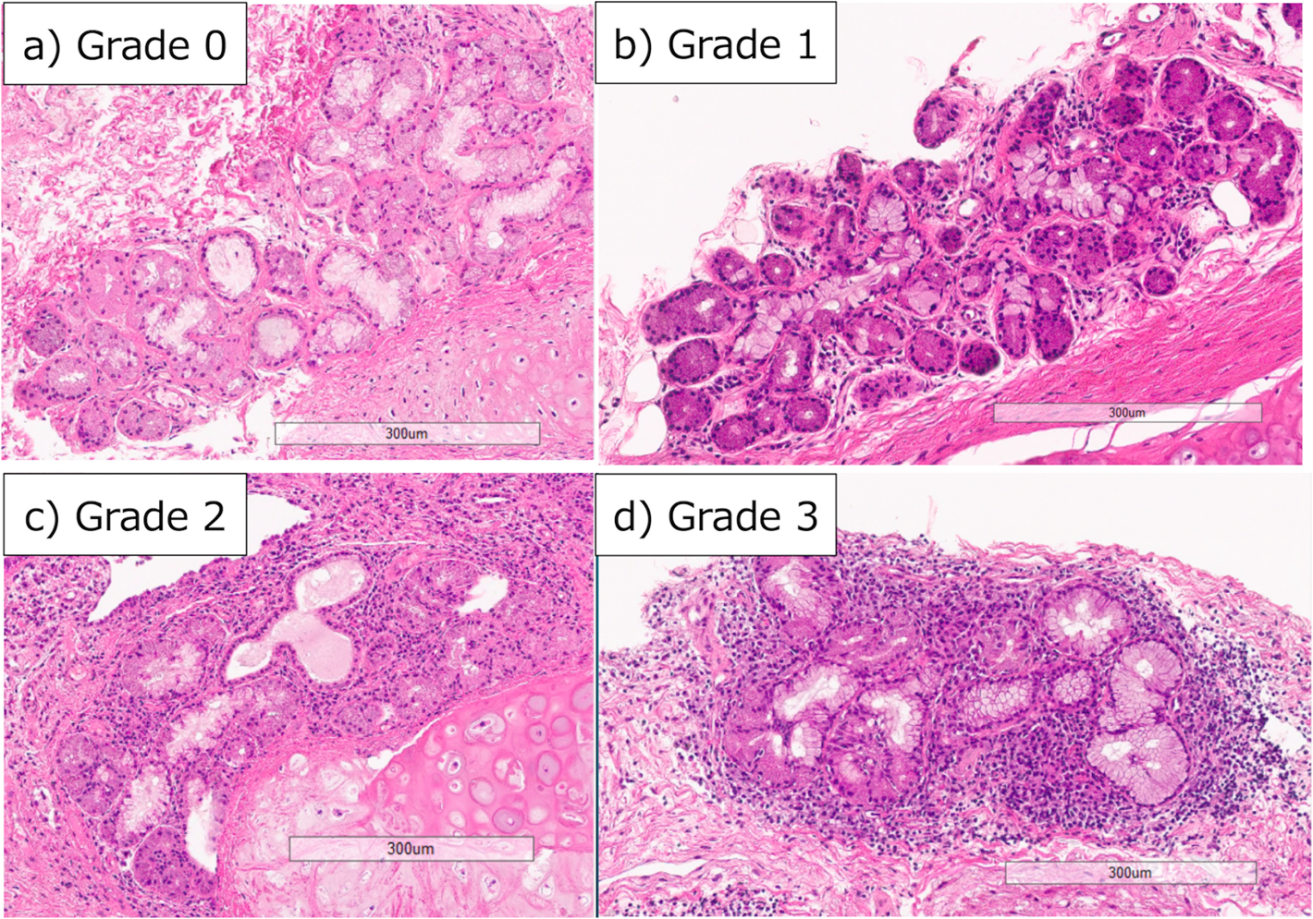
Scoring of lymphocyte and plasma cell infiltration in bronchial gland. **a**; grade 0: absent of lymphocyte and plasma cells infiltration. **b**; grade 1: mild infiltration. **c**; grade2: moderate infiltration with less than 50 lymphocytes and plasma cells. **d**; grade 3: severe infiltration aggregated of 50 or more lymphocytes and plasma cells

Lymphocytes and plasma cells infiltration in bronchial glands were classified into four grades from 0 to 3 (Fig. 2). A “focus” was defined an aggregate of 50 or more lymphocytes and plasma cells. More than one focus infiltration was defined as grade 3. Moderate infiltration less than one focus was defined as grade 2. Grade 1 was defined mild infiltration. Absence of lymphocyte and plasma cell infiltrate was defined as grade 0. The pathologist (T.T: specialized in diffuse lung disease) evaluated without clinical and radiological information.

### Diagnosis of underlying diseases

The diagnosis of idiopathic interstitial pneumonias (IIPs) and chronic hypersensitivity pneumonitis (cHP) was based on consensus using previously reported criteria at a multidisciplinary conference [8–10]. Cases suspected with collagen diseases were consulted with rheumatologists. Patients with SS and systemic sclerosis (SSc) fulfilled the European/American International classification criteria [3, 11]. The diagnosis of dermatomyositis (DM) was based on Bohan and Peter’s [12, 13] and Sontheimer’s criteria [14, 15]. Granulomatosis with polyangitis (GPA) was diagnosed on the basis of 2012 revised International Chapel Hill Consensus Conference Nomenclature of Vasculitides [16]. Mucosa associated lymphoid tissue (MALT) lymphoma was diagnosed in accordance with WHO classification of tumours of haematopoietic and lymphoid tissues [17].

## Results

### Baseline characteristics

**Fig. 3:**
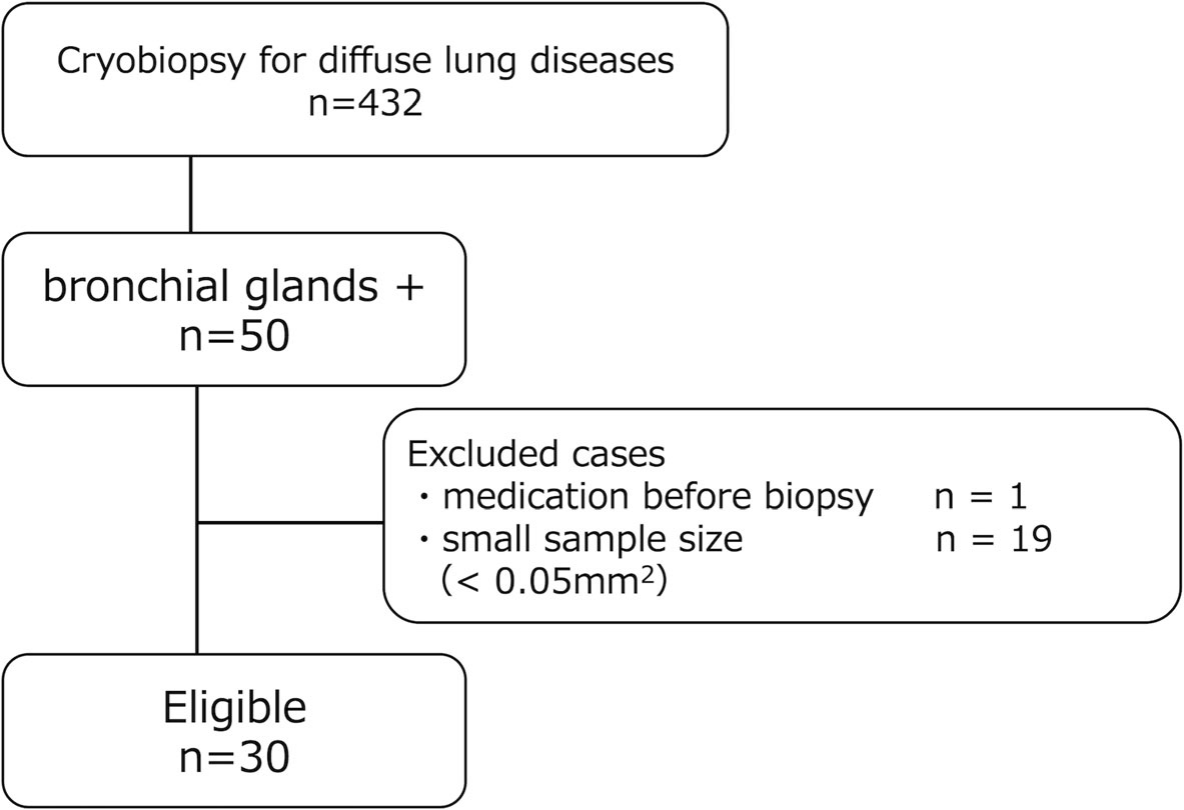
Patients flow diagram

TBLC was performed on 432 cases for diagnosing diffuse lung diseases during the study period. The samples of 50 cases included bronchial glands. Of those, 20 cases were excluded because sample sizes were small or steroid has already been administered before biopsy (Fig. 3). Thirty cases were included in this study. The remaining 30 cases included 17 of idiopathic interstitial pneumonias, 5 of chronic hypersensitivity pneumonia, 6 of connective tissue disease (SS; *n* = 4, systemic sclerosis; *n* = 1, dermatomyositis; *n* = 1) and 2 of other diseases.

The baseline characteristics are shown in Table 1. Median age was 65.5 years. The most frequent underlying disease was 17 of IIPs including 7 of IPF. All cases with SS were primary disease and had no other collagen diseases. The 19 of 30 cases (63.3%) including all SS cases complained cough. Moderate bleeding requiring endobronchial instillation of thrombin was observed in 12.1% of the specimens from which the bronchial gland was biopsied. There was no severe bleeding causing hemodynamic or respiratory instability, requiring tamponade or other surgical interventions, transfusions, or admission to the intensive care unit. There was one case with pneumothorax that did not require drainage.

**Table 1:** Baseline characteristics

Age, years old	65.5(38–80)
Sex (Male/Female), n	10/20
Smoking status (Never/Ex/Current), n	14/14/2
Clinical diagnosis, n	
Idiopathic interstitial pneumonias (IIPs)	17
Idiopathic pulmonary fibrosis (IPF)	7
Nonspecific interstitial pneumonia (NSIP)	2
Cryptogenic organizing pneumonia (COP)	1
Unclassifiable idiopathic interstitial pneumonia (UCIIPs)	7
Chronic hypersensitivity pneumonia (cHP)	5
Connective tissue disease related interstitial pneumonia	6
Sjögren’s syndrome (SS)	4
Systemic sclerosis (SSc)	1
Dermatomyositis (DM)	1
Granulomatosis with polyangitis (GPA)	1
Mucosa associated lymphoid tissue lymphoma	1

Dates are expressed as group median values or numbers of patients

### Scoring of lymphocytes and plasma cells infiltration in bronchial glands

In SS, infiltration of lymphocytes and plasma cells was observed in all cases; grade 1 in one, grade 2 in one, and grade 3 in two cases. In contrast, 11 of 26 in other diseases showed no lymphocytes infiltration, with remaining 15 of grade 1 infiltration (IPF: 6 cases, NSIP: 2 cases, UCIPs: 4 cases, cHP: 2 cases, DM: 1 case). Grade 2 or more infiltration were found only in SS but not in other diseases, while mild lymphocytes infiltration classified as grade 1 were observed in the diseases other than SS. Two SS patients had duct dilation of bronchial glands. There was no case of grade 0 in SS (Table 2).

**Table 2:** Histopathological findings of bronchial glands

Clinical diagnosis	No. of Patients	lymphocytes and plasma cell infiltration Grade 0/1/2/3	No. of Duct dilation
IPF	7	1 / 6 / 0 / 0	0
NSIP	2	0 / 2 / 0 / 0	0
COP	1	1 / 0 / 0 / 0	0
UCIIPs	7	3 / 4 / 0 / 0	0
cHP	5	3 / 2 / 0 / 0	0
SS	4	0 / 1 / 1 / 2	2
SSc	1	1 / 0 / 0 / 0	0
DM	1	0 / 1 / 0 / 0	0
GPA	1	1 / 0 / 0 / 0	0
MALT lymphoma	1	1 / 0 / 0 / 0	0

*IPF* idiopathic pulmonary fibrosis, *NSIP* nonspecific interstitial pneumonia, *COP* cryptogenic organizing pneumonia, *UCIIPs* Unclassifiable idiopathic interstitial pneumonia, *cHP* chronic hypersensitivity pneumonia, *SS* Sjögren’s syndrome, *SSc* systemic sclerosis, *DM* dermatomyositis, *GPA* granulomatosis with polyangitis, *MALT* mucosa associated lymphoid tissue

### Bronchial glands of Sjögren’s syndrome

**Fig. 4:**
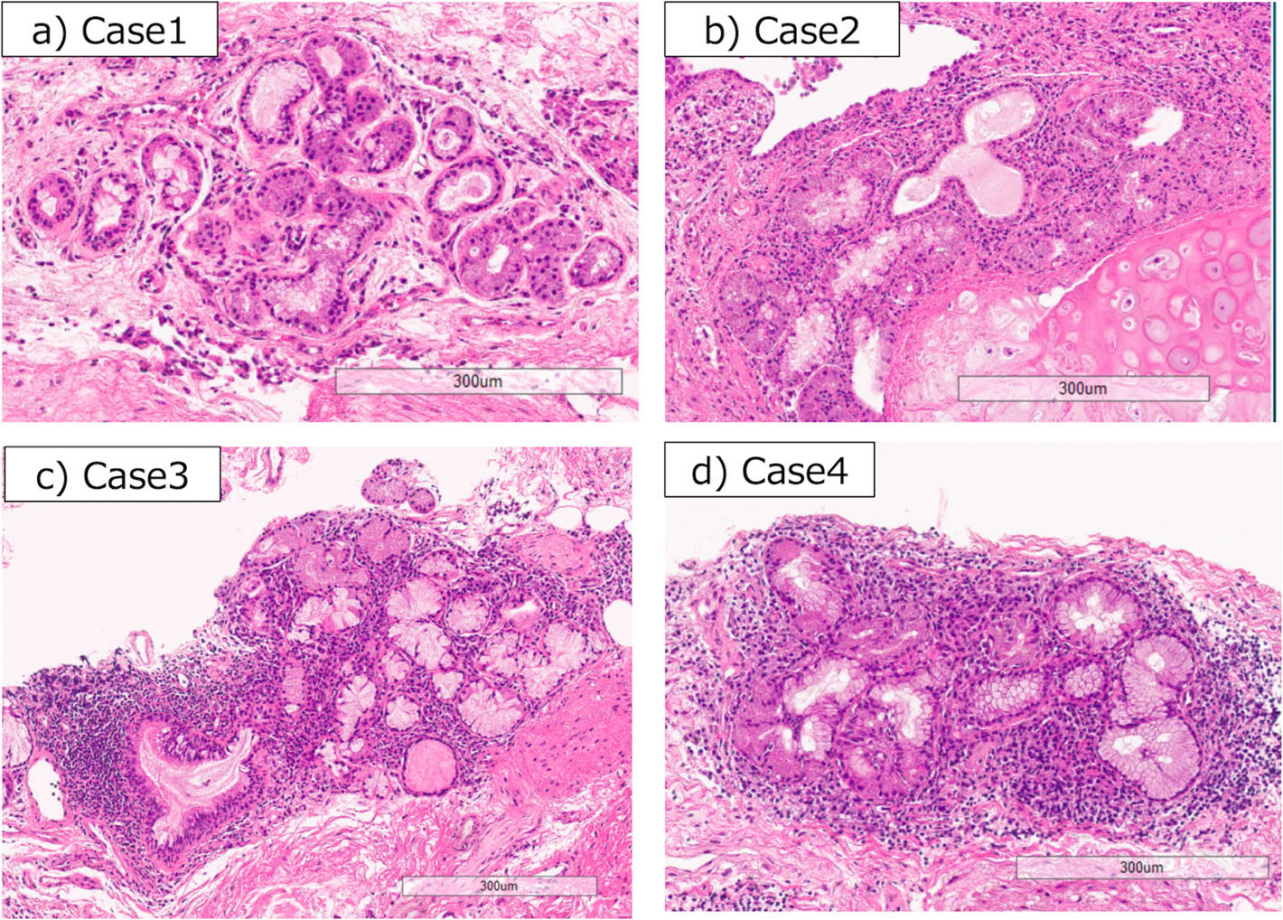
Bronchial glands of Sjogren’s syndrome. **a** Case 1 with grade 1 lymphocyte and plasma cell infiltration and duct dilation. **b** Case 2 with grade 2 lymphocyte and plasma cell infiltration and duct dilation. **c**, **d** Case 3and 4 with grade 3 lymphocyte and plasma cell infiltration

The baseline characteristics are shown in Table 3. All cases were female and positive for anti-SS-A/Ro antibody. Three of the four SS patients had xerostomia or xerophthalmia. Case 1 did not suffer from xerostomia and xerophthalmia. This case was performed a salivary gland biopsy and other cases were diagnosed by other tests that met the diagnostic criteria. Figure 4 shows the bronchial glands of all 4 cases with SS. The bronchial glands of case 3 and 4 revealed high-grade lymphocytic infiltration. Case 2 represented grade 2 lymphocytes infiltration and duct dilation. Case 1 had mild lymphocytes infiltration and duct dilation.

**Table 3:** Baseline characteristics of Sjögren’s syndrome patients

Case	1	2	3	4
Age (years)	70’s	40’s	70’s	70’s
Gender	Female	Female	Female	Female
Smoking status	Never	Never	Ex	Ex
Clinical manifestations				
Cough	+	+	+	+
Sputum	–	+	–	–
Dyspnea	–	+	+	+
Xerostomia	–	–	+	+
Xerophthalmia	–	+	+	+
Anti-nuclear antibody	80 (centromere)	640 (speckled, cytoplasmic)	1280 (homogenous)	1280 (speckled)
Anti-SS-A/Ro antibody	> 240	> 240	> 240	> 240
Anti-SS-B/La antibody	Negative	> 320	Negative	270.1
Pulmonary function				
FVC % pred	90.9	72.1	63.9	66.4
FEV1% pred	90.3	79.4	65.5	73.3
FEV_1_/FVC ratio	77.2	91.4	81.4	86.7
DL_CO_ % pred	97.7	40.1	48.3	52.6
HRCT pattern	NSIP	UIP + NSIP	UIP	NSIP+OP
Salivary gland biopsy	grade 3^a^	N/A	N/A	N/A
Bronchial gland				
Histopathology grading	1	2	3	3
Duct dilation	+	+	–	–

^a^Chisholm-Mason score

*HRCT* high-resolution computed tomography, *N/A* not available

## Discussion

In this study, we examined pathological characteristics of the bronchial glands collected by TBLC. Our results showed that high-grade lymphocytic infiltration in bronchial glands was a distinct characteristics in SS, although mild lymphocytes infiltrations in bronchial glands were occasionally observed in other collagen diseases or other interstitial lung diseases.

SS is characterized by B-cell hyperactivity and lymphocytic infiltration of exocrine glands and other target organs. The pulmonary manifestations of SS are xerotrachea, airway abnormalities, interstitial pneumonia, and lymphoproliferative disorders [18–23]. Xerotrachea is associated with lymphocytic inflammation and atrophy of the submucosal gland [24]. There is only one literature that reported on a case that transbronchial forceps biopsy specimen showed a dense infiltrate of lymphocytes around the bronchial gland in SS patient, despite that the collected bronchial gland was small [25]. Others reported that the bronchial glands in SS showed significant hyperplasia, without mentioning the inflammatory cells in the bronchial glands [26]. As they studied on the autopsy lungs, their subjects may have been affected by treatment such as steroids and immunosuppressant.

SS has characteristic microscopic findings involving lymphocytic infiltration surrounding the excretory ducts in combination with the destruction of acinar tissue. In early stage or advanced phase of SS, there is often slight or none lymphocyte infiltration in the salivary glands. Dilatation of intralobular and interlobular duct is a common finding in the salivary glands of SS, regardless of the degree of lymphocyte infiltration [27]. In this study, duct dilatation in the bronchial glands was observed only in SS. Because this study was a small and retrospective study, we could not compare the degree of respiratory symptoms such as cough with the degree of lymphocytic infiltration of the bronchial glands. In patients with Sjögren’s syndrome who complain of severe persistent cough despite mild or no interstitial pneumonia, exocrine dysfunction of the bronchial glands may be involved, and a bronchial gland biopsy may prove this. Comparing the degree of lymphocytic infiltration of the bronchial glands with clinical symptoms such as cough is future work.

Cryobiopsy is a new technique for diagnosing diffuse parenchymal lung disease [4–7]. TBLC provides larger samples than transbronchial forceps biopsy and more proximal portion of the lung apart from the pleura than SLB. We actively perform TBLC in patients with interstitial pneumonia associated collagen vascular diseases to rule out complications of other diseases such as chronic hypersensitivity pneumonia, to select therapeutic agents, and to predict treatment response and prognosis. A bronchial gland is rarely biopsied by SLB for diagnosing interstitial lung disease. In previous reports on bronchial glands, the specimens were obtained by autopsy or lung resection for localized pulmonary lesions [26, 28]. Autopsied lungs are affected by treatment during their lifetime. There was no report that examined lymphocytes infiltration in bronchial glands of collagen diseases or interstitial lung diseases by lung resection. As we excluded the cases that had been treated before biopsy, the cases of this study were not affected by treatment such as steroids or immunosuppressant. The present study is the first report focusing on lymphocytes infiltration in the bronchial glands with various interstitial lung diseases that were not affected by treatment. Complications of bleeding in the specimens including bronchial glands were not more common than previous reports, and there was no severe bleeding [6].

This study has several limitations. First, this was a small, retrospective study, which may have been subject to various biases. Second, bronchial glands were incidentally biopsied in this study. As we performed TBLC for the diagnosis of diffuse lung diseases, we did not intend to biopsy bronchial glands. Bronchial glands are present in trachea and bronchus with cartilage. If we evaluate focus on lymphocytes infiltration in bronchial glands, transbronchial biopsy in the central airway should be considered. Third, an adequate sample size to evaluate lymphocyte infiltration of bronchial glands is unknown. Because lymphocytes infiltration in salivary glands of SS have irregular distribution, a sufficient volume of specimen is needed in salivary gland biopsy. If a sample is small, the degree of lymphocytes infiltration may be misinterpreted. In this study, we examined the cases that had sufficient size of bronchial glands. We excluded the cases with small size of bronchial glands (< 0.05mm^2^), although it is necessary to examine whether this criteria is appropriate in the future. Finally, we were not able to compare the degrees of lymphocytic infiltration in the salivary glands and the bronchial glands of SS and did not evaluate SS patients without interstitial pneumonia.

## Conclusion

Our results showed that mild lymphocytic infiltration is a nonspecific finding that is also seen in other diseases, but high-grade lymphocytic infiltration of bronchial glands is a distinct characteristics in SS.

## Data Availability

The dataset supporting the conclusions of this article is presented within the article. The detailed clinical data is not available because of patients’ confidentiality.

## Abbreviations

SS: Sjögren’s syndrome
TBLC: Transbronchial lung cryobiopsy
SLB: Surgical lung biopsy
IIPs: Idiopathic interstitial pneumonias
cHP: Chronic hypersensitivity pneumonia
SSc: Systemic sclerosis
DM: Dermatomyositis
GPA: Granulomatosis with polyangitis
MALT: Mucosa associated lymphoid tissue
IPF: Idiopathic pulmonary fibrosis
NSIP: Nonspecific interstitial pneumonia
COP: Cryptogenic organizing pneumonia
UCIIPs: Unclassifiable idiopathic interstitial pneumonia
